# Development of trimester-specific reference intervals for thyroid hormones based on real-world data using a maximum likelihood method

**DOI:** 10.1093/labmed/lmaf053

**Published:** 2025-11-12

**Authors:** Xiaoyan Chen, Yinrui Zou, Yunxia Chu, Zhaoqi Gu, Xiangrong Yang, Yuqiang Huang, Zhaoxi Wang

**Affiliations:** Department of Laboratory Medicine, Linyi Maternal and Child Healthcare Hospital, Luozhuang District, Linyi City 276016, Shandong Province, China; Havy International (Shanghai) Ltd, Yangpu District, Shanghai 200092, China; Department of Laboratory Medicine, Linyi Maternal and Child Healthcare Hospital, Luozhuang District, Linyi City 276016, Shandong Province, China; Department of Laboratory Medicine, Linyi Maternal and Child Healthcare Hospital, Luozhuang District, Linyi City 276016, Shandong Province, China; Department of Laboratory Medicine, Linyi Maternal and Child Healthcare Hospital, Luozhuang District, Linyi City 276016, Shandong Province, China; Department of Pediatric Cardiology, Linyi Maternal and Child Healthcare Hospital, Linyi City 276016, Shandong Province, China; Beth Israel Deaconess Medical Center, Harvard Medical School, Boston, MA, United States

**Keywords:** maximum likelihood method, reference intervals, indirect method, trimester-specific thyroid hormones

## Abstract

**Introduction:**

We sought to establish trimester-specific reference intervals of thyroid-stimulating hormone (TSH) and free thyroxine (FT_4_) using real-world data.

**Methods:**

Deidentified data for FT_4_, TSH, and anti–thyroid peroxidase antibody (TPO-Ab) associated with pregnancies from July 1, 2014, to December 31, 2019, were extracted from the institutions’ medical records. After data cleaning, trimester-specific reference intervals were established using the maximum likelihood method in TPO-Ab–negative pregnancies.

**Results:**

We included 55 323 records after data cleaning. Reference intervals for TSH and FT_4_ built using the maximum likelihood method in the first, second, and third trimesters were 0.40 to 4.09, 0.57 to 4.04, 0.73 to 4.07 mIU/L and 12.2 to 20.5, 10.2 to 18.2, and 9.0 to 15.5 pmol/L, respectively. Compared with reference intervals from the *Guidelines of the American Thyroid Association for the Diagnosis and Management of Thyroid Disease During Pregnancy and the Postpartum* (2nd edition), the maximum likelihood method–calculated reference intervals of FT_4_ were comparable with guideline-suggested reference intervals, with the first trimester reference intervals slightly lower and the second trimester reference intervals showing narrower limits. For TSH, the maximum likelihood method–calculated reference intervals were narrower than guideline-suggested reference intervals.

**Discussion:**

Trimester-specific reference intervals of TSH and FT_4_ for pregnancies were established using the maximum likelihood method. Compared with guideline-suggested reference intervals, no clinically significant discrepancies in FT_4_ and narrower limits in TSH were observed.

## Introduction

Profound physiologic changes in thyroid function occur during pregnancy, including a decrease in thyroid-stimulating hormone (TSH) in the first trimester due to rapidly elevated human chorionic gonadotropin and an increase in TSH-binding globulin concentrations.^1^ Overt hyperthyroidism or hypothyroidism have both been associated with an increased risk of various adverse maternal and child outcomes, including preterm delivery, intrauterine growth retardation, miscarriage, hypertensive disorders, and fetal mortality.^2^ It is critical to maintain adequate thyroid function during pregnancy, and the reference intervals of thyroid hormones are crucial for clinicians to identify hyperthyroidism or subclinical hyperthyroidism and to make clinical decisions. Serum TSH and free thyroxine (FT_4_) vary by region, ethnicity, sex, gestational age, and immunoassay. Therefore, it is essential to establish population-based and pregnancy-specific reference intervals.

As recommended by the Clinical and Laboratory Standards Institute, the direct method for establishing reference intervals requires a strict and random selection of at least 120 reference individuals for every subgroup, which is too costly to accomplish for most laboratories, especially for a test with many subgroups.^3^ It is therefore common to adopt the reference intervals established by manufacturers or large institutions, even though they usually do not work well for local populations. A survey of 490 laboratories in China showed that 90.3% of laboratories use the reference intervals of TSH and FT_4_ provided by the manufacturer, whereas only 3.6% of laboratories use their own reference intervals.^4^

Indirect methods for establishing reference intervals aim to identify the distribution of a nonpathologic population from real-world mixed data, and then establish reference intervals, which is economical and easy to implement for most laboratories. In particular, it makes it possible to establish reference intervals for special groups, such as newborns and older adults, or special specimens, such as cerebrospinal fluid and joint fluid, for which it is typically difficult to get ethical approval for reference individuals. The earliest indirect method was proposed by Hoffmann in 1963, and later, the Bhattacharya method was developed based on normal distribution.^5,6^ Recently, research on indirect methods has gradually increased, including the maximum likelihood (ML) method, the truncated ML (TML) method, the Kolmogorov-Smirnov (Kosmic)–based method, the truncated minimum ꭓ^2^ method, and the refineR method, and these methods have been applied to different items.^7-11^ The ML, truncated ML, Kosmic, truncated minimum ꭓ^2^, and refineR methods were all based on the assumption that the main part of the dataset contains only nonpathologic values and that the proportion of physiologic samples in the dataset can be modeled with a parametric distribution, but they adopt different methods to maximize the likelihood that the observed counts in the histogram representation can be explained by the estimated model and ensure robustness of the optimization procedure. Currently, however, there is no agreed-upon standard by which to evaluate the performance of these methods.^9^

This single-center study aimed to establish trimester-specific reference intervals for TSH and FT_4_ from the data collected from the clinical laboratory of the largest maternal and child health care hospital in Linyi, China, using indirect methods. Among available indirect methods, ML most consistently recovers the correct values from random number simulations and can fit skewed distributions without the use of normalizing transformations. Because ML estimates, although sophisticated in their methodology, have been implemented in the R statistical environment (R Foundation for Statistical Computing), they are reasonably easy to use.^7^ Moreover, these R packages are capable of simultaneously fitting multiple modes, and there is no need to assume that the underlying distributions are normal. Thus, we selected and implemented the ML method in this study, which is one of the indirect methods recommended by International Federation of Clinical Chemistry and Laboratory Medicine.^12^

## Methods

### Study population and selection of samples

**Figure 1.**
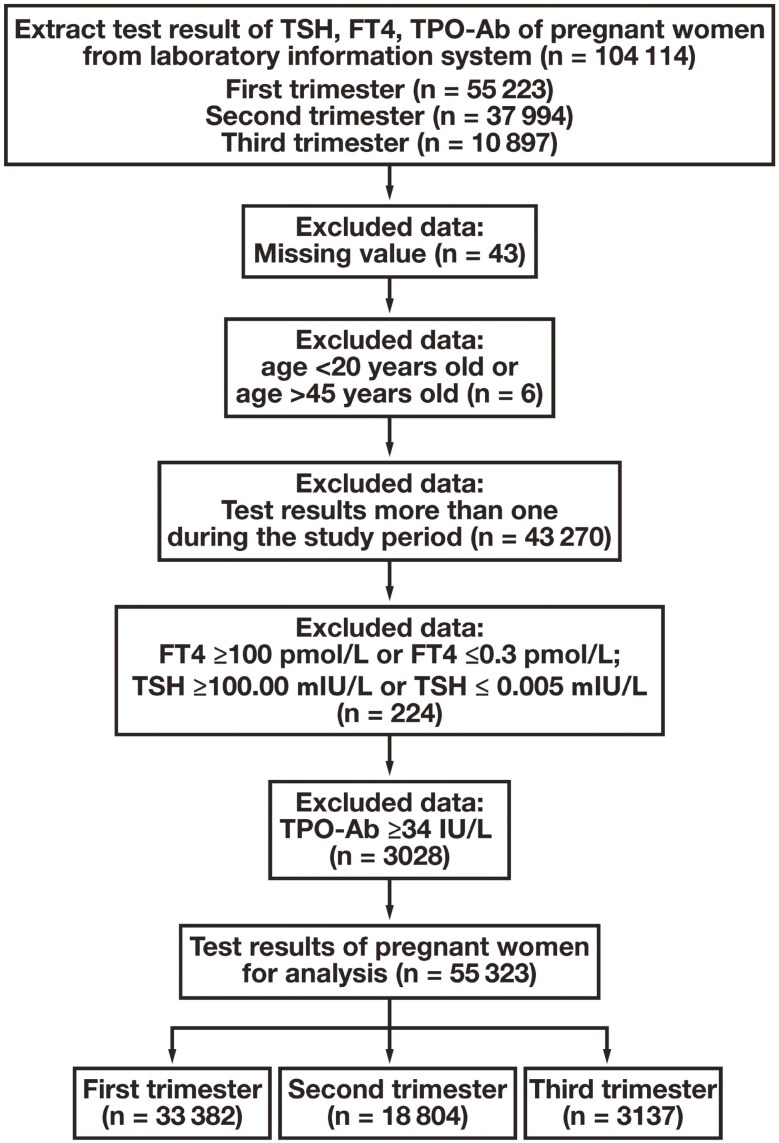
Process of data cleaning. FT4 indicates free thyroxine; TPO-Ab, anti–thyroid peroxidase antibody; TSH, thyroid-stimulating hormone.

This single-center, retrospective study was conducted at Linyi Maternal and Child Healthcare Hospital, Shandong, China, one of the largest hospitals by number of annual live births in China. The study was approved by the Ethics Committee of the study center (No. KYL-2020001). The population consists primarily of individuals from the Han ethnic group. Anonymous results of TSH, FT_4_, and thyroid peroxidase antibody (TPO-Ab) tests were retrieved from the central clinical laboratory between July 2014 and December 2019. We used the R statistical program to perform the exclusion and clearance procedures for extraction of nonpathologic data (Figure 1). Briefly, the exclusion criteria were as follows: (1) missing values; (2) patient age missing; (3) sex is not female; (4) patient is older than 45 years or younger than 20 years of age; (5) more than 1 set of test results during the study period; (6) TSH or FT_4_ values exceeded the analytical measurement range of the testing system (TSH >100.00 mIU/L or <0.005 mIU/L; FT_4_ >100.00 pmol/L or <0.3 pmol/L); and (7) TPO-Ab >34 IU/ml. If any of the exclusion criteria was met, all data from the same serum were excluded.

### Instruments and reagents

Blood samples were collected in the morning and placed in vacuum tubes without anticoagulant, without mandatory fasting for pregnant women. After centrifugation at 2500*g* for 10 minutes within 1 hour after collection, serum was separated and stored at 2 to 8°C if analysis was not performed within 4 hours. All samples were analyzed within 24 hours after collection.

Measurements were performed on an automated analyzer cobas e 601 (Roche Diagnostics) in accordance with the manufacturer’s instructions. Serum TSH, FT_4_, and TPO-Ab were measured using electrochemiluminescence immunoassays with the Roche Elecsys TSH assay, FT_4_ assay, and TPO-Ab assay, respectively. A double-antibody sandwich method was used to test serum TSH (analytical measurement range, 0.005-100.00 mIU/L), and competitive immunoassay methods were applied to test serum FT_4_ (analytical measurement range, 0.3-100 pmol/L) and TPO-Ab (analytical measurement range, 5-600 IU/ml). During the study period, the testing systems did not change, and all new batch reagents were calibrated using Roche’s calibrator. Each batch of tests was carried out when the internal quality control (QC) was acceptable (QC material for TSH and FT_4_: Lyphochek Immunoassay Plus Control [Bio-Rad Laboratories]; QC material for TPO-Ab: Randox IA Specialty Control [Randox Laboratories Limited]). For TSH and FT_4_ assays, the interassay coefficient of variation (s/x̄) × 100 (CV) was less than 8.33%, accounting for one-third of the total allowable error specified by the National Health Commission Clinical Laboratory Center; the intra-assay CVs were less than 6.25%, representing one-quarter of the total allowable error set by the Center. For TPO-Ab, the interassay and intra-assay CVs were less than 10.0% and 7.5%, respectively, accounting for one-third and one-quarter of the quality requirements established by our laboratory. In addition, our laboratory had conducted annual external quality assessments supervised by National Center for Clinical Laboratories to guarantee the accuracy of the test results.

### Reference interval establishment methods and statistical analysis

The ML method was used to fit the model for establishing the reference interval.^13^ The clinical laboratory data were a combination of pathologic and healthy participants. Initially, a smoothed kernel density function was estimated for the distribution of the mixed data. It was assumed that the main part of the distribution of all data represented the healthy population. The distribution of the nonpathologic values was modeled using an assumed statistical model. The parameters of the assumed probability distribution were estimated using the ML estimation, regardless of the distribution used initially. The Kolmogorov-Smirnov statistic was used to detect the main part of the data. Next, lower and upper reference limits of the reference intervals were calculated as the 2.5 and 97.5 percentiles of the estimated distribution for the nonpathologic value.^12^ Gestational trimesters were defined as follows: the first trimester was 4 to 12 weeks of gestation, the second trimester was 13 to 27 weeks of gestation, and the third trimester was beyond 27 weeks of gestation. Reference intervals were established for each trimester.

Normal distribution is the most widely used distribution in ML estimation, but better results can be obtained if more suitable distributions are used. In our dataset, the distribution of FT_4_ was approximately normal. Because the distribution of TSH was skewed, the γ distribution was integrated to model a range of items due to its flexible shape, which could capture various statistical distributions without a prior data transformation to a normal distribution.

Based on the abnormal rate of pregnancies and degree of cleaning of the research data, we estimated the ratio of the healthy to pathologic population as an initial fitting parameter. According to the fitting process of ML estimation, however, minor variation in the estimated ratio did not change the fitting parameters. Thus, we did not need to artificially select the best fitting parameters accurately before fitting the model.

The R *mixdist* package was employed to fit the model.^13^ It permits the use of γ distribution, which can both exhibit skewing distribution and approximate the normal distribution. All computational analyses were performed using R, version 4.0.1, statistical software (supplemented with the *dplyr, mixtools*, and *mixdist* packages). All figures were produced using the R *ggplot2* package. Pregnant women in each trimester were allocated to 1 of 5 age groups: 20 to 25 years, 26 to 30 years, 31 to 35 years, 36 to 40 years, and 41 to 45 years. The Kruskal-Wallis test was used to compare the difference across the 5 age groups. The comparison of abnormal rates between 2 reference intervals was assessed using the ꭓ^2^ test. *P* < .05 was considered statistically significant.

## Results

A total of 104 114 records of pregnant women who had undergone TSH, FT_4_, and TPO-Ab testing during pregnancy between July 1, 2014, and December 31, 2019, were retrieved from the local medical laboratory. After applying the exclusion and inclusion criteria (Figure 1), 55 323 individuals were enrolled, with a sequential decrease over trimesters, including 33 382 patients in the first trimester, 18 804 in the second trimester, and 3137 in the third trimester. The age and trimester distributions are presented in Table 1.

**Table 1.** Population Distribution, by Age and Trimester

		Trimester		
Age, y	No. (%)	First	Second	Third
20-25	12 010 (22)	7555 (23)	3844 (20)	611 (19)
26-30	24 556 (44)	14 793 (44)	8421 (45)	1342 (43)
31-35	12 886 (23)	7603 (23)	4498 (24)	785 (25)
36-40	4715 (9)	2727 (8)	1675 (9)	313 (10)
41-45	1156 (2)	704 (2)	366 (2)	86 (3)
Total	55 323 (100)	33 382 (100)	18 804 (100)	3137 (100)

**Figure 2.**
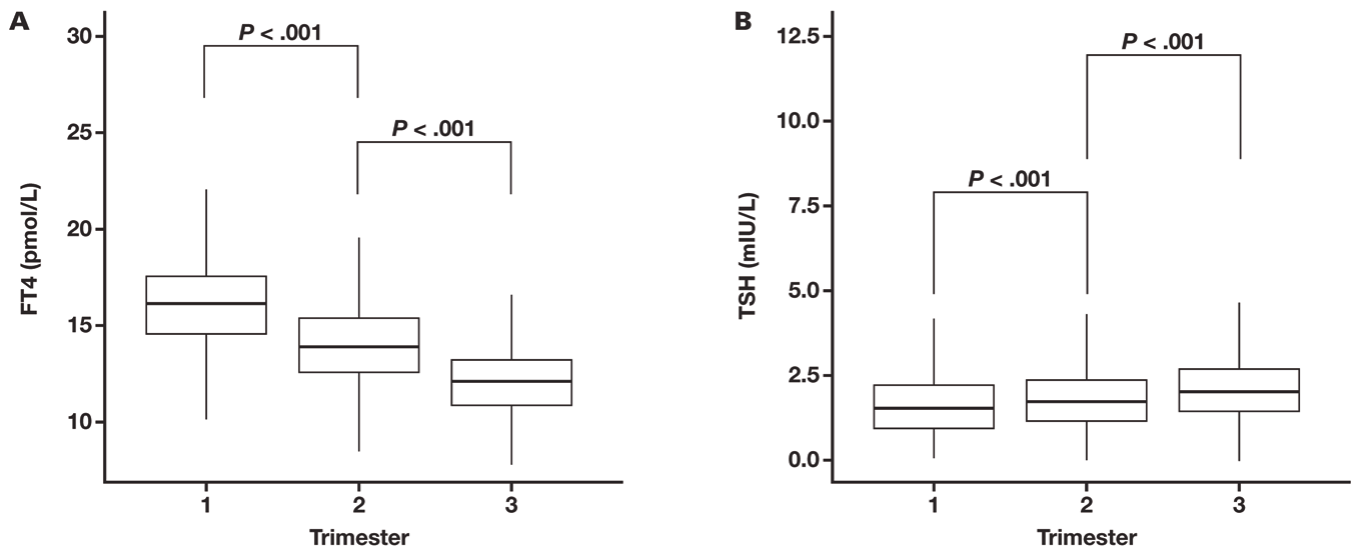
(**A**) Median difference in FT_4_ levels across 3 trimesters; (**B**) median difference of thyrotropin across 3 trimesters. FT_4_ indicates free thyroxine; TSH, thyroid-stimulating hormone.

We saw a trimester-specific decrease in FT_4_ levels (*P* < .001) but a trimester-specific increase in TSH levels (*P* < .001) (Figure 2). Within each trimester group, there was statistical significance among age groups for both TSH and FT_4_ (Table 2).

**Table 2.** Age-Specific Reference Intervals of FT_4_ and TSH

		First trimester		Second trimester		Third trimester	
Hormone	Age group	Median (range)	*P* value	Median (range)	*P* value	Median (range)	*P* value
FT_4_, pmol/L	20-25	16.31 (12.5-20.8)	<.05	14.34 (10.8-18.6)	<.05	12.49 (9.3-16.3)	<.05
	26-30	16.09 (12.5-20.3)		13.90 (10.4-18.2)		12.00 (9.1-15.5)	
	31-35	15.86 (12.1-20.1)		13.64 (10.0-18.1)		11.89 (9.0-15.3)	
	36-40	15.54 (11.8-19.9)		13.19 (9.8-17.3)		11.52 (8.7-14.9)	
	41-45	15.36 (12.0-19.3)		12.98 (9.4-17.4)		11.58 (9.0-14.7)	
	All ages	16.00 (12.2-20.5)		13.90 (10.2-18.2)		12.00 (9.0-15.5)	
TSH, mIU/L	20-25	1.66 (0.47-4.05)	<.05	1.81 (0.56-4.23)	<.05	1.97 (0.74-4.14)	<.05
	26-30	1.62 (0.47-3.90)		1.81 (0.61-4.03)		1.97 (0.72-4.20)	
	31-35	1.62 (0.45-3.98)		1.73 (0.56-3.97)		2.01 (0.73-4.31)	
	36-40	1.58 (0.48-3.73)		1.58 (0.44-3.87)		1.87 (0.61-4.26)	
	41-45	1.67 (0.60-3.61)		1.61 (0.47-3.87)		1.95 (0.70-4.18)	
	All ages	1.59 (0.40-4.09)		1.77 (0.57-4.04)		1.95 (0.73-4.07)	

Abbreviation: FT_4_, free thyroxine; TSH, thyroid-stimulating hormone.

**Figure 3.**
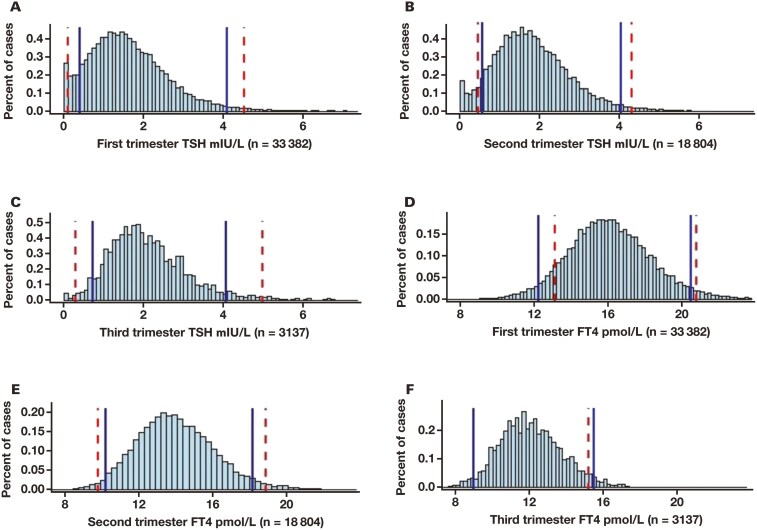
The vertical blue lines indicate the reference intervals that were built based on our laboratory data. The vertical red lines indicate the reference intervals recommended in the *Guidelines of the American Thyroid Association for the Diagnosis and Management of Thyroid Disease During Pregnancy and the Postpartum*. (**A, B, C**) The reference intervals of thyrotropin in each of 3 trimesters. (**D, E, F**) The reference intervals of FT_4_ in each of 3 trimesters. FT_4_ indicates free thyroxine; TSH, thyroid-stimulating hormone.

We then compared 95% trimester-specific reference intervals of TSH and FT_4_ built from the ML method with those suggested in *Guidelines of the American Thyroid Association for the Diagnosis and Management of Thyroid Disease During Pregnancy and the Postpartum* (2nd edition) (hereinafter referred to as the “Guidelines”) for laboratories using the Roche assay system (Figure 3). For TSH, the ML-calculated trimester-specific reference intervals were obviously narrower than the Guidelines-suggested reference intervals for Roche assay systems, especially for the second and third trimesters. The ML reference intervals had a relatively stable upper limit for all trimesters, whereas the Guidelines-suggested reference intervals upper limits increased as pregnancy preceded.

From early to late pregnancy, the ML-calculated and Guidelines-suggested reference intervals for Roche analysis of FT_4_ were gradually shifted to lower values. In general, the ML-calculated t**r**imester-specific reference intervals for FT_4_ were comparable with Guidelines-suggested reference intervals, with the first trimester ML-calculated reference intervals slightly shifted to lower values; the second trimester ML-calculated reference intervals had a slightly narrower limits, and the third trimester reference intervals had a higher upper limit (Figure 3).

For FT4 and TSH levels of pregnant women included in this study, we calculated the abnormal rates using Guidelines-suggested reference intervals and ML-calculated reference intervals in this study. There were significant differences between the two reference intervals for all trimesters (TABLE 3).

## Discussion

In this study, we established trimester-specific reference intervals for TSH and FT_4_ using the ML method on real-world medical records. Compared with the Guidelines-suggested reference intervals for Roche analyzers, our results showed that FT_4_ reference intervals were comparable, but TSH intervals were statistically significantly different. During pregnancy, there was an increased trend for TSH but a decreased trend for FT_4_. Within each trimester, the FT_4_ and TSH levels for pregnant individuals decreased as maternal age increased.

Compared with the Guidelines-suggested reference intervals for pregnancies, the TSH ML-calculated reference intervals were narrower. Especially in the third trimester, the upper limit of our study was statistically significantly lower than that suggested in the Guidelines. For FT_4_ reference intervals, although the lower and upper limits of ML-calculated reference intervals were close to those suggested in the Guidelines, there were minor differences in each trimester. We hypothesize that the following reasons might explain these differences. Firstly, the populations were different. The diversities in geographic location, living habits, and climate might have contributed to the discrepancy. Second, because the iodine nutrition status of the pregnancies in our study was unknown, iodine-deficient individuals were not excluded from the analysis, which might be attributed to the difference in reference intervals. Third, the different sample sizes might have influenced the establishment of reference intervals. The reference intervals of this study were established from data from 58 323 pregnancies, which was much larger than the sample size in the research mentioned in the Guidelines using the direct method to calculate reference intervals. Fourth, different test schemes lead to different compositions of real-world data. At the study center, a unified testing strategy was adopted—that is, TSH, FT_4_, and TPO-Ab were tested simultaneously for pregnant women with unknown thyroid function status. If there were any positive finding, further thyroid-related tests would be performed. In other research, thyroid hormone tests can be performed using different schemes. When only TSH was tested, if there was an abnormal result, an FT_4_ test would be ordered. In some cases, TSH, FT_4_, and free triiodothyronine tests could be ordered only if there was no abnormal result. The different composition of real-world data derived by various test procedures might cause differences in reference intervals. Fifth, different data-cleaning schemes could lead to differences in the reference intervals established. In our data-cleaning scheme, only pregnant women who had had only 1 thyroid hormone test in 1 trimester during the study period were included. When multiple thyroid hormone tests were ordered in different trimesters for a pregnant woman, then all test data for that individual were deleted. According to the 2018 *Guideline of Preconception and Prenatal Care*,^14^ only 1 thyroid hormone test should be administered in the first trimester. In our hospital, if the test results were missing for the first trimester, the test was administered in the subsequent trimester. If the first test result was abnormal, the test was repeated and other tests were ordered. Based on the administration procedure and our data-cleaning scheme, the test results in the second or third trimester could be retained only if the tests were ordered for the first time. Therefore, there were fewer data in the third trimester in our research. It was suggested that TPO-Ab–positive pregnant women should be excluded when establishing reference intervals because TSH levels are higher in TPO-Ab–positive pregnancies.^15,16^

More importantly, various distributions of gestational weeks among pregnant women require different reference intervals. During pregnancy, TSH and FT_4_ levels change dramatically and rapidly, especially in the first trimester. Ideally, reference intervals should be established in different groups when heterogeneity is obvious, but most studies have established trimester-specific reference intervals, ignoring the heterogeneity of data in each trimester. These studies on thyroid hormone reference intervals did not indicate the ratio of pregnancies in each gestational week, but gestational week distributions varied across studies.^17-19^ For example, Yu et al^19^ selected pregnancies from the 10th to 14th week of gestation as the first trimester, from the 20th to 24th week of gestation as the second trimester, and from the 30th to 34th week of gestation as the third trimester. In another study, Zhang et al^20^ took the 8th to 12th week of gestation as the first trimester, the 16th to 20th week of gestation as the second trimester, and the 28th to 36th week of gestation as the third trimester. For the study by Huang et al,^17^ the researchers established the reference intervals of the first trimester with pregnancies in the 7th to 12th week of gestation, while Guo et al^18^ included pregnancies from the 4th to 12th week of gestation. Thus, various distributions of gestational age could cause the wide variations in reference intervals, which should be given particular attention in clinical practice.

The maternal age–associated decrease in FT_4_ level throughout all trimesters was consistent with most previous reports of thyroid hormone changes during pregnancy.^21^ Comparing pregnant women under 30 years of age with pregnant women 30 years of age or older, Diéguez et al^22^ did not find differences in mean FT_4_ levels in the first or second trimester. The different results in that study might be explained by a different ethnic population from northern Spain without exclusion of abnormal TPO-Ab findings as well as grouping the participants into only 2 age groups divided at 30 years of age.^22^ In our research, pregnant women in each trimester were allocated to 1 of 5 age groups set at 5-year intervals, with statistical significance among age groups. The clinical explanation of thyroid hormone levels in pregnant women is unclear, however, and needs further research.

There are heterogeneous results from previous studies on the age-associated changes of thyrotropin. Similar to our findings, Du Yunwang et al^23^ reported a decreasing trend in thyrotropin values with age. On the contrary, some studies reported age-associated increases,^24-26^ while other studies found no age-related change.^22,27^ The sample size of our study (58 323 pregnant women) was much larger than previous research (only 9770, 1477, and 477 pregnancies were included in the above-mentioned studies, respectively).^25,26,28^ In addition, the demographic differences, such as age, gestational age, TPO-Ab status, and iodine deficiency, could also contribute to various observations.

Moreover, few previous studies on pregnancy-specific reference intervals of thyroid hormones included women of advanced maternal age, and these sample sizes were often small. Advanced maternal age is a risk factor for high rates of maternal and neonatal complications.^28^ Thyroid function screening for pregnancies at advanced maternal age should receive more attention, and dedicated reference intervals should be established for local pregnancies.

Our study has some limitations. First, the iodine nutrition status of pregnant women in the study center could not be extracted and analyzed. Second, the sample size of women in the third trimester was small. Finally, we had only a small number of pregnant women older than 35 years of age. More pregnant women with advanced maternal age should be included in pursuing reference intervals adjusted for iodine nutrition status in future research.

We used the ML method to establish the reference intervals of TSH and FT_4_ for pregnant women in our research center. It is recommended that each laboratory establish reference intervals for thyroid hormones suitable for the local population to optimize the management of thyroid function during pregnancy. In addition, attention should be paid to the distribution of gestational age when establishing reference intervals for thyroid hormones due to dramatic changes in thyrotropin and FT_4_ levels throughout pregnancy. Continuous reference intervals are recommended.

**Table 3.** Abnormal Rates of TSH and FT_4_ Calculated According to Reference Intervals from Guidelines and This Research

Trimester	Hormone^a^	Abnormal rate with reference intervals of Guidelines, %	Abnormal rate with reference intervals of this research, %	*P* value
First	TSH	5.75	12.79	<.001
	FT_4_	11.39	8.00	<.001
	TSH and/or FT_4_	15.56	17.46	<.001
Second	TSH	9.27	12.07	<.001
	FT_4_	4.06	6.37	<.001
	TSH and/or FT_4_	11.67	16.04	<.001
Third	TSH	4.19	11.43	<.001
	FT_4_	7.92	6.03	.0001
	TSH and/or FT_4_	11.09	16.38	<.001

Abbreviations: FT_4_, free thyroxine; TSH, thyroid-stimulating hormone; Guidelines, *Guidelines of the American Thyroid Association for the Diagnosis and Management of Thyroid Disease During Pregnancy and the Postpartum* (2nd edition).

^a^For TSH, the reference intervals are 0.09-4.52, 0.45-4.32, and 0.30-4.98 mIU/L in the first, second, and third trimesters, respectively. For FT_4_, the reference intervals are 13.15-20.78, 9.77-18.89, and 9.04-15.22 pmol/L, respectively, in the first, second, and third trimesters.
